# CSF Diagnostics: A Potentially Valuable Tool in Neurodegenerative and Inflammatory Disorders Involving Motor Neurons: A Review

**DOI:** 10.3390/diagnostics11091522

**Published:** 2021-08-24

**Authors:** Karsten Krause, Maximilian Wulf, Paula Sommer, Katalin Barkovits, Matthias Vorgerd, Katrin Marcus, Britta Eggers

**Affiliations:** 1 Department of Neurology, Heimer Institute for Muscle Research, University Hospital Bergmannsheil, Ruhr-University Bochum, 44789 Bochum, Germany; karsten.krause@rub.de (K.K.); matthias.vorgerd@rub.de (M.V.); 2 Medizinisches Proteom-Center, Medical Faculty, Ruhr-University Bochum, 44801 Bochum, Germany; maximilian.wulf@rub.de (M.W.); paula.sommer@rub.de (P.S.); katalin.barkovits@rub.de (K.B.); katrin.marcus@rub.de (K.M.); 3 Medical Proteome Analysis, Center for Protein Diagnostics (PRODI), Ruhr-University Bochum, 44801 Bochum, Germany

**Keywords:** cerebrospinal fluid, ELISA, biomarker, amyotrophic lateral sclerosis, spinal muscular atrophy, peripheral neuropathies, Guillain-Barré syndrome, motor neurons

## Abstract

Cerebrospinal fluid (CSF) diagnostics has emerged as a valid tool for a variety of neurological diseases. However, CSF diagnostics has been playing a subordinate role in the diagnosis of many neurological conditions. Thus, in the multitude of neuromuscular diseases in which motor neurons are affected, a CSF sample is rarely taken routinely. However, CSF diagnostics has the potential to specify the diagnosis and monitor the treatment of neuromuscular disorders. In this review, we therefore focused on a variety of neuromuscular diseases, among them amyotrophic lateral sclerosis (ALS), peripheral neuropathies, and spinal muscular atrophy (SMA), for which CSF diagnostics has emerged as a promising option for determining the disease itself and its progression. We focus on potentially valuable biomarkers among different disorders, such as neurofilaments, cytokines, other proteins, and lipids to determine their suitability, differentiating between different neurological disorders and their potential to determine early disease onset, disease progression, and treatment outcome. We further recommend novel approaches, e.g., the use of mass spectrometry as a promising alternative techniques to standard ELISA assays, potentially enhancing biomarker significance in clinical applications.

## 1. Introduction

Cerebrospinal fluid (CSF) diagnostics emerged as a valid tool for a variety of neurological diseases. It is routinely used to detect neuronal diseases such as acute or chronic meningitis or encephalitis and is even able to distinguish between an acute viral and a bacterial intrathecal infection [1,2]. Furthermore, it is an important diagnostic tool for detecting multiple sclerosis and metastasising tumours, as in the case of leukaemia, and tumours of the central nervous system (CNS) [3,4]. In recent years, CSF diagnostics has also been introduced in the field of neurodegenerative and inflammatory diseases such as Alzheimer’s disease (AD) [5,6,7,8,9], Parkinson’s disease (PD) [10,11,12,13,14], and autoimmune encephalitis [15,16,17,18,19,20]. However, CSF diagnostics has also played a subordinate role in the diagnosis of many other neurological diseases. Thus, in the multitude of neuromuscular diseases in which the motor neurons are affected, a CSF sample is rarely taken routinely. However, CSF diagnostics may contribute to the diagnosis and treatment of neuromuscular disorders. In this review, we therefore focused on neuromuscular diseases such as amyotrophic lateral sclerosis (ALS), peripheral neuropathies and spinal muscular atrophy (SMA), for which CSF diagnostics emerged as a promising option for determining the presence of the disease itself and its progression.

## 2. Materials and Methods

A literature search was performed using the Medline and Cochrane databases included in PubMed. As abundance of published articles was highly variable among the discussed neuromuscular disorders, the parameters for study selection were adapted accordingly. For ALS the terms, “ALS” or “amyotrophic lateral sclerosis” and “CSF” or “cerebrospinal fluid” were chosen as a first indication. Additionally, the terms “cytokine” or “neurofilament” or “TDP-43” were added. As for neurofilaments, the number of studies was comparably high (103 publications), so only studies conducted within the last 5 years (from December 2015 to December 2020, 56 publications) containing a high number of patients in the cohorts (>45 ALS patients within the cohort) were used for in-depth review. For all other terms, a search period of 15 years was chosen. We further focussed on studies using similar techniques (ELISA) enabling comparability of studies for different biomarkers. For SMA, the terms “SMA” or “spinal muscle atrophy” or “spinal muscular atrophy” and “CSF” or “cerebrospinal fluid” were chosen. Results were further filtered for studies published in the last 15 years (from January 2006 to May 2021, 147 publications).

For the reviewed peripheral neuropathies, a smaller number of publications was found compared to the literature research on ALS, therefore specific exclusion criteria in terms of study population or publication date weren’t applicable. The terms searched for Guillain-Barré syndrome were “Guillain-Barré syndrome” or “GBS” in combination with “CSF”, “cerebrospinal fluid”, “biomarker”, “neurofilament”, “proteomics”, or “lipidomics”. For multifocal motor neuropathy, the terms “multifocal motor neuropathy” or “MMN” and “CSF”, “cerebrospinal fluid” or “biomarker” were selected. The terms used for Lyme neuroborreliosis were “neuroborreliosis” or “lnb” in combinaton with “CSF”, “cerebrospinal fluid” or “biomarker”. For all studies, only those matching the questions covered in this review were included. Literature search terms and inclusion and exclusion criteria for all neuromuscular disorders discussed in this review are further visualized in a flow diagram (Figure S1).

## 3. Amyotrophic Lateral Sclerosis

ALS is a heterogeneous neurodegenerative disease affecting both upper and lower motor neurons and is marked by a progressive loss of bulbar and limb function. In 5% of all cases ALS is found to be familial. To date over 100 gene mutations and over 30 genes have been associated with different types of familial ALS [21]. However, the majority of ALS cases remain sporadic, in which genetic and environmental factors both play important roles increasing the risk of developing ALS. Still, the identification of risk factors remains a challenging step, as ALS presents itself as a multifactorial and genetically diverse disease with a comparably low incidence rate. Until now, there is no curative treatment option and after the first symptoms present themselves, patients typically die due to respiratory failure within three years [22]. The clinical management relies mainly on symptomatic and palliative care with the aim of maintaining or improving quality of life [23]. To date, no definite diagnostic marker for ALS is available and diagnosis solely relies on excluding potential other causes of progressive motor neuron dysfunction, using the El Escorical criteria [24]. The disease progression is assessed by the ALS Functional Rating Scale [25]. The resulting scores are not only used for diagnosis, but also to predict patient survival. Since the diagnosis of ALS is based mainly on clinical examination, diseases displaying similar symptoms should first be ruled out. Thus, a definitive diagnosis is often delayed and possibly formulated after 9–15 months of disease [22]. Therefore, the need to find definite early diagnostic markers is of utmost importance. 

Diagnostic tools encompass neuroimaging, whereby a routine MRI is conducted. In this context, patients with ALS might show atrophy of motor cortices and degeneration of motor tracts [22,26], but those changes are neither very sensitive nor very specific and are consequently usually used to exclude competing causes. 

In clinical routine, liquid biopsies most often present as valuable tools for disease verification. Commonly, blood, plasma, saliva or CSF samples are used as liquid biopsy options. Since ALS is a neurodegenerative disorder, CSF could be the most suitable liquid biopsy option, as it constantly surrounds the brain tissue. Several studies have already focused on the evaluation of various potential biomarkers detectable in CSF for their ability to reliably differentiate between ALS, various other neurological diseases, so-called ALS mimics, and healthy cohorts [27,28,29,30,31,32,33,34,35,36,37,38,39,40,41,42,43]. The most frequently studied biomarkers in this context include cytokines [30,37,38,39], as the immune system plays a major role in ALS formation. In addition, TDP-43 [31,32,33,34,35], a protein known to hyperphosphorylate and aggregate during ALS progression, and neurofilaments [28,29,36,40,41,42,43], the intermediate filaments of the nervous system that are known to contribute to ALS when failing to organise neuronal integrity, were found to be potentially relevant biomarkers. With respect to possible CSF diagnostic approaches for ALS, in this review we focussed on these three groups of proteins and assessed current strategies and their outcome to predict ALS disease and progression. 

### 3.1. Cytokines

Cytokines comprise a large group of small, secreted proteins, including interleukins, chemokines, interferons, and tumor necrosis factor. They function as elementary mediators in inflammatory processes and are secreted by various immune cells like T and B lymphocytes, endothelial cells, mast cells, and macrophages [44,45,46], especially during neuroinflammation. Microglia account for 5–10% of cells in the brain [47] and play an indispensable role in the production of pro- and anti-inflammatory substances [48]. In ALS, activated microglia appear in regions that are significantly affected by degeneration of motor neurons as well as in areas that contain only weak damage [49]. Therefore, the role of microglia in ALS is still under debate [50]. However, other cytokine producing cells also show enhanced levels in CNS human tissue in ALS. The spinal cord, in particular, exhibits intense cell infiltration of T cell lymphocytes [51], mast cells [52], and dendritic cells [53]. In addition, the well-established superoxide dismutase (SOD1) mouse model of ALS provides evidence that inflammation is highly involved in ALS pathogenesis through characteristic elevated levels of several cytokines [54,55,56]. Interestingly, studies showed that SOD1 mutant microglia cells in particular produce more toxic substances than wild-type microglia, among them TNF-α [57,58]. Furthermore, CSF and blood/serum samples of ALS patients likewise contain dysregulated cytokine levels [30]. Since CSF reflects CNS metabolism, CSF analysis represents a significant source for various investigations. Based on the evidence that inflammatory processes and the resulting dysregulation of cytokines play a major role in ALS, many studies have investigated cytokine concentrations in CSF and serum in ALS in search of potential biomarkers. In this regard, elevated levels of interleukin-6 (IL-6) [59], monocyte chemoattractant protein-1 (MCP-1), granulocyte colony-stimulating factor (G-CSF) [60] and prostaglandin E2 (PGE2) [61] have already been reported in CSF. In this review, we will focus on four major studies examining cytokine and chemokine level in CSF from large cohorts of ALS patients and controls. A detailed description of every study discussed here, involving cohort selection size and composition, body fluid, as well as used technique/antibody and detected cytokine levels can be found in Table S1. As an overview, the results of cytokine evaluations are further summarized in Table 1.

Tateishi et al. investigated cytokine concentration in the CSF of patients with sporadic ALS (sALS), lower motor neuron disease (LMND), and of a control group with non-inflammatory neurological diseases (ONDs) [37]. The ALS cohort always comprised patients classified depending on their diagnosis into clinically definite or clinically probable. In addition, patients were characterized by different onsets of ALS pathogenesis, like bulbar, trunk, upper, and lower limb onset. For LMND and sALS, disease severity related to both progression and development were determined using the ALSFRS-R score. As a control, the OND group consisted of patients with e.g., cervical spondylosis, lumbar herniation, sporadic and hereditary spinocerebellar atrophy. For the detection of 27 different cytokines in CSF, Tateishi et al. used the multiplexed fluorescent bead-based immunoassay. They were able to identify increased levels of fifteen cytokines in sALS compared to ONDs (G-CSF, VEGF, CCL2, CCL4, CCL5, CCL11, CXCL8, CXCL10, TNF-a, IFN-y, IL-1β, IL-7, IL-9, IL-12, IL-17). Among them, some could be related to clinical parameters. CCL2 and CXCL8 showed a negative while VEGF and CCL4 revealed a positive correlation with the ALSFRS-R score. CXCL10 and CCL4 exhibited a negative correlation with disease progression rate and a positive correlation with disease duration. However, it was pointed out that positive correlations of CCL2 with disease progression, which was also found to be apparent in a previous study [60], was absent in the current study, probably due to the study group composition. Moreover, CCL2 and CXCL8 displayed a positive and VEGF a negative correlation with total CSF protein level. Thus, the authors suggested that increasing CSF protein levels are linked to blood–brain barrier (BBB) damage. The assumption that BBB permeability increases in context of ALS pathogenesis has already yielded in many other studies [62,63,64] Interestingly, Tateishi et al. found no significant differences between LMND and OND as well as between LMND and sALS. 

Another study by Mitchell et al. also applied the multiplex bead-based immunoassay for analysis of 27 cytokines and growth factors in CSF [38]. In relation to the previous study from Tateishi et al., the cytokine panel differed slightly. Here, the control group also contained individuals with ONDs. In addition to multiplex cytokine bead assay, an enzyme-linked immunosorbent assay (ELISA) was used for determination of transferrin and β2-microglobulin in CSF. To investigate the frequently postulated association between *H36D* polymorphism in the hemochromatosis gene (*HFE*), all patients in this study were also genotyped for *H36D* and *C282Y HFE*. Results showed that all cytokines except for IL-1 were detected. In addition, thirteen differential cytokines, among them eleven increased in ALS and two in OND, were observed (IL-6, GM-CSF, IL-2, IL-15, IL-17, CCL4, FGF basic, G-CSF, VEGF, CCL3, CCL2; IL-10, IFN-y). Those with the most significant *p*-value were IL-6, GM-CSF, IL-10, IL-2, and IL-15. The elevated values of IL-17, CCL4, G-CSF, VEGF, and CCL2 are consistent with the results of the previous study. Surprisingly, the five markers with the most significant *p*-values do not show any correspondence. Authors pointed out that some of these markers are involved in microglia pathways, lymphocyte activation, and cell proliferation, therefore confirming the association between ALS pathogenesis and CNS inflammation. However, for systematic analysis of the correlation between *HFE* variants and CSF cytokine profiles, all individuals were grouped based on their genotypes (homo- and heterozygotes were clustered as one). *H63D HFE* variation is highly associated with familial ALS and, similarly to *C282Y HFE*, causes cellular iron accumulation. Patients carrying an *H63D HFE* variant compared to wild type controls showed enhanced concentrations of β-2 microglobulin and CXCL8. In contrast, subjects with the *C282Y HFE* allele demonstrated elevated levels of IL-7, IL-12, and PDGF bb. Interestingly, CXCL8, IL-7 and IL-12 exhibited elevated levels in ALS compared to OND in the study reported by Tateishi et al., therefore the relation between *HFE* variants remains unclear.

A further study by Furukawa et al. evaluated the cytokine profiles of patients with progressive muscular atrophy (PMA), ALS, and multifocal motor neuropathy (MMN) [30]. Due to the fact that all three diseases share similar pathological features and are difficult to distinguish from each other, misdiagnosis often occurs [65,66]. Thus, the purpose of this study was to investigate potential differences in CNS inflammation between MMN, PMA, and ALS. A group of patients with ONDs was used as control. Through the assumption, that PMA is often considered a variant of ALS, authors reviewed and compared clinical data from both groups. Criteria for differentiation of ALS from PMA included the presence of upper motor neuron (UMN) signs and symptoms, electrophysiologic indications of LMN involvement, no conduction block, disease duration under five years, and, especially for ALS, the El Escorial criteria. Furukawa et al. emphasized that the clinical profiles of PMA and ALS do not overlap and thus are comparable. For their approach they also used a multiplex bead array assay and thereby discovered 27 cytokines in serum and CSF samples. Additionally, for detection of soluble TNF receptor (TNFR1), an ELISA kit was used. Association of CSF cytokine profiles with clinical data showed that IL-4 and CCL11 appeared to be associated with a lower ALSFRS-R score. In contrast, IL-10 demonstrated a correlation with an elevated ALSFRS-R score, indicating presence of mild symptoms. Results of CSF cytokine profiles showed increased levels of seven cytokines in ALS compared to ONDs: CCL11, IL-17, PDGF-BB, G-CSF, FGF-2, IL-4, and IL-7. Both previous studies were also able to determine enriched values of IL-17, G-CSF, and IL-7. Concentrations of IL-17 and G-CSF measured by Tateishi et al. and Furukawa et al. were highly variable (IL-17 varies between 2.7 +/− 0.194 and 32.1 +/− 54.0, G-CSF between 9.670 +/− 0.484 and 27.6 +/− 43.8), whereas those of IL-7 seemed to be very stable (1.495 +/− 0.075 and 1.6 +/− 1.9). In contrast, PDGF-BB and IL-4 were also measured within the previous studies but showed elevated levels in the present implementation only. FGF-2 and CCL11 were also measured in all observations but could be identified as elevated in ALS in just two of them. While VEGF was found by Tateishi et al. and Mitchell et al. as significantly increased in ALS, in this study VEGF showed predominantly elevated values in PMA, yet also slightly enhanced in ALS. CCL4, CCL3, and CCL2 were reported to be significantly changed in the study by Mitchell et al., and CCL2 and CCL4 were also changed in Tateishi et al. However, in the previous comparison, no increase was found to be specific to ALS. Additionally, comparison with other motor neuron disease illustrates that the majority of cytokines regulated in ALS react similarly in patients with PMA. To conclude, the authors highlighted that PMA and ALS showed similar CSF cytokine profiles making them difficult to distinguish from each other.

The approach of Italiani et al. focused on specific analysis of the interleukin-1 family in ALS. In general, the IL-1 family comprises eleven cytokines, including receptor antagonists and pro- and anti-inflammatory molecules [39]. Here, the study investigated four IL-1 family mediators (IL-1β, IL-18, IL-33, IL-37) and their endogenous inhibitors (IL-1Ra, sIL-1R2, IL-18BP, sIL-1R4) in serum and CSF. Overall, the cytokine panel is quite different from the other studies mentioned here. IL-1β and IL-1Ra are the only ones that were also measured by the previous studies, but only IL-1β was described in one study as significantly changed in ALS. Italiani et al. highlighted that to evaluate a cytokine’s biological effect, it is essential to measure its inhibitor, since only the free form remains active. Clinical data were used from 125 CSF samples from 54 sporadic ALS patients. In this study, the ALSFRS-R score was also used for clinical assessment of ALS patients. Serum (*n* = 40) and CSF (*n* = 65) samples from individuals with non-inflammatory diseases were used as controls. Here, ELISA was used exclusively as a cytokine assay. Based on results of this study, it appears that many interleukins and their inhibitors, such as IL-1β, IL-37, IL-1Ra, and IL-36 were often not detectable in all samples. A significant increase was only detected for IL-18 (*p* < 0.0001) and IL-18BP (*p* < 0.0001) in serum of sALS. The authors linked the elevated IL-18 levels to the inflammasome complex and accordingly to caspase-1 activity, which is as well increased in SOD 1 mouse model of ALS. The fact that increased caspase-1 activity is usually accompanied by an enhanced level of IL-1β, which is missing in this study, was therefore described by the authors as a more local tissue-related outcome and therefore not detectable in body fluids. The lack of detection of some cytokines and their inhibitors in serum or CSF underlines this assumption. 

In conclusion, a transferable and distinct CSF or serum cytokine analysis in ALS appears to be quite challenging. Through comparison of the studies mentioned here, it becomes evident that many of the measured cytokines do not always appear to be differentially regulated. In addition, the findings by Furukawa et al. [30] pointed out that many of the cytokines that emerged as markers for ALS reveal similar activity in patients with PMA [67,68,69]. Therefore, it is crucial to identify potential biomarker candidates. In the case of a few selected cytokines, such as G-CSF or IL-17, the same pattern can be observed consistently. Detailed information can be found in Table 1. This table also allows the recognition of several interesting cytokine expression patterns; IL-1ra shows no significant differences between ALS and controls, but in the comparison between inflammatory diseases, such as MMN, GBS, or LNB and controls. IL-13 alone showed a significant difference in LNB compared to controls and MS, while in the other comparisons no significant difference was found. G-CSF displayed significant differences in the comparison of the degenerative diseases and LNB, whereas in the comparison of the demyelinating diseases no changes were detected. These findings suggest distinct roles of specific cytokines in the different diseases, potentially leading to unique answers of the immune system. For further studies and comparative results, common cytokine panels, utilization of related study cohorts, and additional comparison with other neurodegenerative diseases will be necessary to elucidate the involvement and specificity of cytokines in ALS pathogenesis. 

### 3.2. TDP-43

TDP-43 (TAR DNA-binding protein 43) is a 414-amino acid nuclear protein that is highly conserved across species and is ubiquitously expressed in tissues, including heart, lung, liver, spleen, kidney, muscle, and brain [70]. Its physiological functions are highly versatile as it binds to single strand DNA, RNA, and proteins to regulate transcription, translation, mRNA transport and stabilization [71,72]. In addition TDP-43 is further capable of assembling into stress granules, indicating its protective role against cell stress [73]. On the other hand, pathological hyper-phosphorylated and ubiquitinated TDP-43 was found to deposit as inclusion bodies in the brain and spinal cord of patients with the motor neuron disease ALS and frontotemporal lobar degeneration (FTLD) [74]. In fact, numerous mutations in the TDP-43 encoding gene *TARDBP* have been found to be associated with ALS and FTLD [75], mainly leading to an increased protein aggregation, enhanced cytoplasmic mislocalization, altered protein stability, and a stronger resistance to proteolytic breakdown. A detailed description of pathological mechanisms induced by the misfolding of TDP-43 can be found in the following review and will not be further discussed here [76]. In addition to mutations in the TDP-43 encoding gene, numerous other gene mutations have also been linked with ALS [76,77,78,79,80,81,82]. Although the pathological hallmark of TDP-43 aggregation is commonly associated with ALS and FLTD, TDP-43 positive inclusions have been observed in a variety of neurodegenerative disorders [83,84,85]. Recently, effort has been made to verify if TDP-43 may serve as a potential biomarker candidate for ALS, potentially facilitating the currently complicated diagnosis. Quantification of TDP-43 levels in CSF from patients with ALS, FTLD, and various other neurological disorders was achieved using different biochemical techniques resulting in contradictory recommendations on whether TDP-43 may serve as a biomarker for the diagnosis of ALS and differentiating it from other neurological diseases. A detailed description of cohort selection size and composition, body fluid, as well as used technique/antibody and detected TDP-43 levels can be found in Table 2 and Table S2.

Most commonly, ELISA was used for TDP-43 concentration determination. Early studies initially focussed on whether the concentration of TDP-43 in CSF of healthy individuals differed significantly from ALS patients [32]. Kasai et al. pointed out that, as a group, ALS patients showed significantly higher CSF levels of TDP-43 than age-matched controls. However, levels of TDP-43 reached an upper confidence level beyond 95% in only six ALS patients out of 30 (20%). All six patients were examined within 10 months of disease onset, all other patients instead were examined after 11 months from the onset of illness. This leads to the suggestion that TDP-43 levels in CSF may mainly be increased in early stages of ALS. 

A second study referring to the first one described above set out to determine if TDP-43 levels were able to distinguish between ALS and other common neurodegenerative disorders, such as Parkinson’s disease, multiple sclerosis and Guillain-Barré syndrome (GBS) [31]. Using the same ELISA method, Noto et al. were able to verify that CSF TDP-43 levels in ALS patients were significantly higher than for all other neurodegenerative disorders analysed (ROC analysis showed a sensitivity of 59.3% and a specificity of 96.0%). Interestingly, no significant differences were found in CSF TDP-43 levels among the different neurodegenerative disorders. The authors of this study were not only able to verify that the level of CSF TDP-43 is a factor in distinguishing ALS from other neurological diseases but were also able to establish a dependency between survival rate and the level of TDP-43. The survival time of patients with CSF TDP-43 levels > 27.9 ng/mL (*n* = 12) was longer than for those patients with CSF TDP-43 levels < 27.9 ng/mL (*n* = 15) from the time of CSF collection, leading to an independent prognostic factor of survival. Authors additionally stated that age, site of onset, gender and disease duration were not significantly related to survival time. Therefore, the hypothesis stated by Kasai et al. that early onset ALS patients may exhibit a higher concentration of TDP-43 in CSF could not be confirmed in the second study. 

A third study aimed to differentially diagnose ALS patients from GBS by quantitative determination of TDP-43 in the CSF, again using an ELISA-based approach [33] (detailed information on cohort selection size and composition, as well as used technique/antibody and detected TDP-43 levels can be found in Table 2). As a proof of principle, Hosokawa et al. used supernatant of cultured cells transfected with various TDP-43 constructs to confirm the ELISA assay. In the subsequent analysis of the patient samples, they were able to clearly establish that the concentrations of TDP-43 in the CSF of ALS patients were significantly higher than those of patients with GBS, reaching a sensitivity of 71.4% and a specificity of 84.6%. Additionally, they were able to confirm that there was almost no association between TDP-43 levels and age in either group.

As an alternative to ELISA, TDP-43 specific immunoblotting was also conducted [35], enabling the use of different TDP-43 antibodies: monoclonal TDP-43 antibody specific for amino acids 205 through 255, polyclonal antibody against the N-terminal amino acids 6 through 24, and polyclonal antibody against the C-terminal amino acids 396 through 414 of TDP-43. In this study, patients were either diagnosed with ALS or FLTD, a highly similar neurodegenerative disorder with several common hallmarks, or a combined diagnosis of ALS and FLTD and compared to a control group (detailed information on cohort selection size and composition, as well as used technique/antibody and detected TDP-43 levels can be found in Table 2). With their multi-antibody-based approach, Steinacker et al. confirmed that the polyclonal TDP-43 antibodies recognized a 45 kDa band in all analyzed samples. The two monoclonal and N-terminus–specific antibodies, however, did not detect any specific bands, but C-terminus–specific antibodies detected the 45 kDa band and additional bands at approximately 20 kDa in all CSF samples. Relative quantification of 45 kDa bands revealed significant differences among the diagnostic groups and again no correlation between patient age and 45 kDa TDP-43 levels could be detected supporting the ELISA-based analyses. Authors pointed out, that there was a wide variation of TDP-43 levels among all CSF samples (*n* = 53) analysed. TDP-43 levels ranged from 7% to 164% (median = 60%) in the ALS group, 26% to 92% (median = 63%) in the FTLD group, 9% to 105% (median = 24%) in the ALS plus FTLD group, and 5% to 79% (median = 28%) in the control group. Two of three patients in the ALS plus additional signs of frontal disinhibition (DI) group had low TDP-43 levels (16% and 17%), whereas one patient had a level of 100%. Nevertheless, statistical analysis revealed significant differences among all tested groups (*p* = 0.046), resulting in the hypothesis that TDP-43 levels were increased in the ALS and FTLD groups compared with controls (*p* = 0.03 and *p* = 0.02, respectively).

A second study utilizing Western immunoblotting for TDP-43 identification stated that free TDP-43 may only have a limited role as a diagnostic tool and suggested isolating exosomes out of CSF samples as an additional source for a potentially accurate TDP-43 concentration analysis. In the described study, Feneberg et al. investigated combined CSF and serum samples, blood lymphocytes, brain material, and purified exosomes from CSF for TDP-43 by one- (1D), and two-dimensional (2D) Western immunoblotting (WB) and targeted mass spectrometry (multiple reaction monitoring (MRM)) in patients with ALS, FTLD and, non-neurodegenerative diseases [34]. CSF/blood ratios of TDP-43 were used to determine whether TDP-43 is mainly blood derived taking CSF/blood ratios of similar molecular weight as control. WB analysis clearly detected a 45 kDa band by N-terminal TDP-43 antibody in CSF and serum from patients and controls. In addition, two bands of higher intensity in the range of 50 kDa and 55 kDa were detected. Confirmation of specific binding was achieved using a C-terminal antibody against detecting the specific 45 kDa band in CSF samples. Strikingly, similar signal intensities for TDP-43 in serum and CSF could be observed. In addition, brain material of ALS patients with positive TDP-43 pathology was assessed. A positive signal at around 45 kDa and 50 was detected, while no pathological 20 kDa could be observed in contrast to the study discussed before. 2D WB analysis of samples additionally offered the possibility to detect post-translational modifications, as they play a major role in TDP-43 aggregation. Here, similar spot patterns in the CSF and serum of the ALS patients, but also in those of the control patients, were found. Post-translational modifications of TDP-43 were found to be similar in CSF, whole blood, and blood lymphocytes. In the brain material with TDP-43 pathology instead an additional higher spot pattern at about 50 kDa shifted to a more acidic isoelectric point (pI), potentially representing the phosphorylated TDP-43. As no pathological alteration could be confirmed in CSF, the authors assumed that the majority of CSF TDP-43 may not the pathologically altered. Nevertheless, no overall quantitative assessment of WB intensities was used for concentration determination of TDP-43. Instead, exosomes isolated out of CSF from ALS patients and controls were used to determine concentrations of non-blood-derived TDP-43. TDP-43 could be identified via WB in purified exosomes irrespective of diagnostic groups. Mass spectrometric MRM analysis of exosome derived TDP-43 resulted in no significant differences between the diagnostic groups. Still, authors stated that the established MRM technique has to be further refined and optimized with regard to pathologically altered TDP-43.

In summary, TDP-43 cannot yet be utilized as a biomarker for the differentiation and diagnosis of ALS compared to other neurological diseases and healthy individuals. On the one hand, the different ELISA- and immunoblot-based studies show high variations in the CSF TDP-43 detected concentrations, which may be caused by interfering high abundant proteins such as immunoglobulins and albumin hampering a good antibody binding efficiency [86]. On the other hand, no pathological forms of the TDP-43 protein were routinely included in the analyses. One way to optimise and validate the currently available assays would be to improve antibody specificity. Additionally, the currently existing assays should be established for pathologically relevant forms of TDP-43, by specifically enriching the hyperphosphorylated form. Further targeted mass spectrometry could be a promising method, as it could not only determine the absolute concentration of TDP-43 in various body fluids and tissues, but also determine the ratio of pathological TDP-43 and the non-phosphorylated form.

### 3.3. Neurofilaments

To date, neurofilament light chain (NfL) and phosphorylated neurofilament heavy chain (pNfH) are identified as the most promising candidate biomarkers for predicting ALS. In general, neurofilaments (Nfs) represent intracellular intermediate filaments in the central and peripheral nervous system. In neurons, their highly elastic abilities contribute to control axonal diameters, ultimately resulting in the modulation of neuronal response to stimuli [87]. Nf protein assemblies can include several subunits, among them the NfL, the neurofilament medium chain (NfM) and the NfH. Disruption of the Nf organization was found to be one of the key characteristics of many neurological conditions [88,89,90]. Therefore, in recent years emerging evidence suggested that the abundance of NfL and pNfH in CSF and plasma correlates with ALS [43,91,92,93,94,95]. Hence, various studies focused on determining whether both Nfs might inherit a potential as ALS biomarkers. In this review, we will focus on recent studies conducted in the last 5 years, analyzing large ALS cohorts with a focus on CSF. All studies focused on determining NfL and pNfH concentration via ELISA assays, several using assays provided by the same vendors enabling a good starting point for the direct comparison of derived data. Detailed information on cohort selection size and composition, body fluid, as well as used technique/antibody and detected NfL and pNfH levels among the assessed studies can be found in Table 3 and Table S3.

Poesen et al., and Rossi et al., both evaluated large ALS patient cohorts alongside neurological disease controls and partially ALS disease mimics [29,41]. Nevertheless, Poesen et al., included ALS patients harboring a mutation in known ALS-causing genes, such as *C9orf72*, *SOD1*, *FUS* and *TARDBP*. Both studies used identical ELISA assays for the subsequent concentration determination of NfL and pNFH. Both studies independently detected a significantly higher concentration of NfL and pNfH in ALS patients ranging from 4700 pg/mL in [41] to 9427 pg/mL in [29] for NfL and 1700 pg/mL in [41] and 2366 pg/mL in [29] for pNfH. However, Rossi et al. stated that only the comparison between ALS and CTRL 1 (includes patients with ALS-mimic diseases) reached significance for pNfH. Poesen et al. subsequently stated that pNfH levels were found to be superior to differentially diagnose ALS patients from disease mimics resulting in a sensitivity of 90.7% (CI 84.9%–94.8%) and a specificity of 88.0% (CI 75.7%–95.5%). They could further predict that CSF pNfH and NfL were able to discriminate patients with fast from those with slow disease progression, albeit with rather poor performance characteristics.

Delaby et al. instead solely focused on determining the concentration of NfL using the same kit as described in the previous two studies. Again, NfL levels of ALS patients were compared with a variety of other neurological disorders (see Table 3) [40]. NfL levels again were found to be the highest in ALS patients detecting an NfL concentration of 2953 pg/mL. They could additionally conclude that CSF levels were positively correlated with age and were associated with sex.

Besides the commercially available ELISA kits, Olsson et al. designed an in-house ELISA using 2 NfL monoclonal antibodies [42]. They were able to determine NfL concentrations of ALS patients as well as healthy controls and patients with a variety of neurological disorders (Table 3). Again, they could conclude that NfL concentrations were highest in participants with ALS with a median (range) of 4185 (2207–7453) pg/mL, followed by patients suffering from FTD with a median (range) of 2094 (230–7744) pg/mL, both reaching statistical significance. Olsson et al. further correlated their NfL derived data with TDP-43 load in 17 brain regions in the 60 patients from whom data were available. Here, they were able to find a positive, statistically significant correlation between CSF levels of NfL and TDP-43 load in 13 of 17 brain regions (76.4%).

An ambitious study evaluating the comparability and reproducibility of NfL and pNfH determination via ELISA assay carried out by Oeckl et al. set out to test ALS patient and control CSF samples from 15 different centers in a multicenter study [28]. All samples were analyzed in two different laboratories using identical ELISA kits, providing an elaborate view on data reproducibility among cohorts and laboratories. This paved the way for a clear recommendation, whether an ELISA-based concentration determination of NfL and pNfH may function as a valuable diagnostic marker for ALS. Measured concentrations in ALS patients were comparable between most centers and inter-laboratory variation of measurements was stated to be acceptable for pNfH. Although samples were slightly differently treated in the different centers, a high diagnostic performance was reached for pNfH and NfL, confirming the calculated cut-off value of 568.5 pg/mL, which was similarly achieved in a previous single center study analyzing over 450 ALS patients [36]. The authors stated that the observed consistency of Nf concentrations in ALS patients among all centers indicates that efforts at optimization and standardization of CSF collection and processing results in robust and reproducible results. Nevertheless, authors clearly stated the limitations of their study and several factors needing further optimization, such as sample sizes, different preanalytical conditions, and patient characteristics. Additionally, the authors hypothesized that NfL autoantibodies regularly detected in CSF of ALS patients may potentially lead to inter-individual and between-center variations.

Besides CSF-based studies to elucidate the concentration of NfL and pNfH, the majority of studies focus on determining plasma-derived pNfH concentrations, enabling a less invasive method to predict ALS diagnosis. One particular study we want to focus on in our review is a plasma and CSF combined study conducted by De Schaepdryver et al. The authors examined 85 ALS patients, 215 patients with various other neurological disorders (disease controls, DC), and 31 ALS mimics enlarging the so far conducted studies by a clear recommendation whether NfL and pNfH are able to distinguish between ALS and clinically highly similar disorders, such as Kennedy disease or motor neuropathy [43]. Authors could first determine that NfL and pNfH CSF levels were always identified as being higher than serum levels, reaching a magnitude of over 10 fold. Still, both CSF and serum pNfH concentrations of ALS patients were both found to be significantly increased compared to DC and ALS mimics. Even early stages of ALS could safely be differentiated between the two control groups in serum and CSF. Still, ROC curve analyses showed that the sensitivity and specificity of discrimination was found to be higher for CSF. Hence, the authors concluded, that serum pNfH concentrations showed a larger overlap between patients with ALS and disease controls or ALS mimics than CSF pNfH concentrations. As a consequence, CSF pNfH determination performs better when discriminating between patients with ALS and ALS mimics.

Additionally, studies comparing plasma/serum and CSF levels of either NfL or pNfH came to similar conclusions [92,94]. However, Gong et al., stated that CSF NfL concentration determination could even be replaced by serum-based analyses for the assessment of damage to motor neurons and axons, as their data showed a strong correlation of NfL levels in both body fluids. Other studies claimed that NfL concentrations in plasma and CSF may be superior to determine disease severity, progression, and survival than prognosing ALS in patients [27,96].

Combining hypotheses and results raised and determined in the aforementioned studies, NfL and especially pNfH have been developed as reproducible and sensitive prognostic markers distinguishing ALS from healthy people but also from patients suffering from different neurological disorders and ALS mimics. Still, we have to point out that factors, such as the usage of different body fluids, different cohort compositions, varying protocols for body fluid withdrawal and collection, and patient specific parameters such as mutations and autoantibodies may alter the specificity of the results. Nevertheless, all studies displayed promising and comparable results using ELISA assays for the diagnosis of ALS in large cohort studies.

## 4. Peripheral Neuropathies

Peripheral neuropathies summarize a multitude of diseases that affect peripheral motoric, sensory and/or autonomic nerves. Usually, a distinction is made by the pattern found in nerve conduction studies into axonal or demyelinating neuropathies. Causes can be, for example, metabolic, toxic, inflammatory, hereditary, or paraneoplastic [26]. Due to this variety of causes, an elaborated differential diagnosis is crucial to find a causal therapy. We here present new approaches in CSF-based biomarkers that could ease this differential diagnosis in selected neuropathies.

### 4.1. Guillain-Barré Syndrome

Guillain-Barré syndrome usually presents with an acute ascending palsy and sensory loss, often following respiratory or gastrointestinal infections [26]. The diagnostic is based on typical clinical and electrophysiological findings, as well as the typical CSF finding of cytoalbuminologic dissociation with elevated albumin and a normal cell count. A therapy is usually administered by intravenous immunoglobulins or plasma exchange. Electrophysiological examinations usually show a demyelinating pattern, which is referred to as acute inflammatory demyelinating polyneuropathy (AIDP). In about 5% of cases in European and North American populations, axonal degeneration is the predominant finding [97], which is called acute motor axonal neuropathy (AMAN). Axonal GBS often has prolonged and incomplete recovery. To evaluate clinical severity, usually a Hughes functional score (GBS disability scale score) is used. For example, an F-score of 0 corresponds to healthy, 3 describes the ability to walk 5 m with help and an F-score of 6 refers to death [98]. Another system of clinical evaluation is the Medical Research Council grading system (MRCS), in which muscle strength in upper arm abductors, elbow flexors, wrist extensors, hip flexors, knee extensors, and foot dorsal flexors is graded and summed up by the following system: 0 = paralysis, 1 = trace of muscle contraction, 2 = muscle movement is possible with gravity eliminated, 3 = muscle movement is possible against gravity, 4 = muscle strength is reduced and 5 = normal strength [99]. The Overall Disability Sum Score (ODSS) rates motor function from 0 (no symptoms) to 10 (bound to wheelchair and unable to use both arms) [100].

#### 4.1.1. Neurofilaments

The above mentioned neurofilament light chain (NfL) and phosphorylated neurofilament heavy chain (pNfH) are intracellular proteins that are found in the CNS as well as in the peripheral nervous system. They were not only evaluated as biomarkers in ALS but also in GBS. This circumstance already suggests a possible pitfall that the differentiation of diseases might not be eased by this marker. An overview of the evaluated studies is displayed in Table 4.

Petzold et al. investigated NfH CSF levels in 23 patients with GBS using a standard ELISA [101]. The upper normal level was set with 0.73 ng/mL. The abundance of axonal degeneration was investigated by nerve conduction studies and electromyography. Patients were assigned to two groups according to those findings. CSF NfH levels were compared between patients with axonal degeneration and demyelination. It could be shown that patients with electrophysiological signs of axonal degeneration had a 12.5-fold higher CSF NfH level compared to patients with a demyelinating pattern (1 ng/mL vs. 0.08 ng/mL, *p* = 0.0135). Outcomes were measured using both the Medical Research Council grading system (MRCS) and the F-score. Patients with elevated NfH CSF levels (>0.73 ng/mL) had a worse outcome (F-score ≥ 2 (odds ratio 14.40, 95% CI: 1.38 to 150.81), MRCS ≤ 45 (odds ratio 15.00, 95% CI: 1.33 to 169.87), NfH levels correlated with the final F-score (R = 0.47, *p* = 0.024) and MRCS (R = −0.57, *p* < 0.01).

In a subsequent study by Petzold et al., NfH CSF levels in 38 GBS patients were evaluated by ELISA and compared to levels of patients with other neurological diseases [102]. There was no significant difference in NfH levels between both groups. Patients were grouped by their outcome. NfH levels were higher in the group with a poor outcome of an F-score ≥ 3 (univariate testing *p* = 0.019, multivariate testing *p* = 0.004). NfH levels above the upper normal limit of 0.73 ng/mL therefore predicted a poor outcome (*p* = 0.01, odds ratio 7.3, 95% confidence interval 1.2–46.2).

A study by Wang et al. investigated CSF pNFH levels in the GBS subgroups AIDP (*n* = 11) and AMAN (*n* = 11) and compared them to other neurological diseases (ONDs, *n* = 10) using a commercial sandwich ELISA [103]. CSF levels of pNfH were increased in both subforms of GBS compared to OND (*p* < 0.001). A comparison between AMAN and AIDP showed a significant difference with higher pNfH levels in AMAN (*p* < 0.05). In AMAN, increased levels of pNfH correlated with GBS disability scale scores in the acute phase (*p* = 0.001, R = 0.881), the plateau phase (*p* = 0.0002, R = 0.897), and the recovery phase (*p* = 0.006, R = 0.764).

Dujmovic et al. performed serial spinal taps in three patients and evaluated NfH levels using the identical ELISA [104], as in the abovementioned studies [101,102]. Clinical and electrophysiological data were also acquired. A statistical analysis was not performed, but it seemed that high levels of NfH were associated with a poor outcome.

The approach of Axelson et al. included CSF sampling at the onset of the disease and a clinical follow-up 9–17 years after the disease in 18 patients [105]. Hughes functional score was performed at nadir (the point of lowest performance) and follow-up, but the ODSS was only assessed at follow-up. NfL levels were assessed by an ELISA. GBS patients had a higher NfL level than healthy controls (*p* < 0.0001). Patients that showed a poor outcome at follow-up had higher NfL level than patients with a good outcome. NfL levels over 10000 ng/L had persistent disability with a median ODSS of 5.5, patients below this limit had a median ODSS of 0. NfL levels also correlated with the F-score in the acute phase (*p* = 0.01, R = 0.59). Initial Nf correlated also with the quality of life at follow-up, as measured by the PCS score (*p* < 0.05, R = −0.65).

Mariotto et al. determined NfL levels in CSF and serum with an ELISA in 25 patients with different inflammatory neuropathies including 5 GBS patients [106]. Serum NFL levels were significantly (*p* < 0.001) increased in the neuropathy group (median 31.52 pg/mL, range 4.33–1178) compared to healthy controls (median 6.91 pg/mL, range 2.67–12.78). No significant differences between the different diseases were found. The CSF NfL levels in both groups were unfortunately not mentioned in the paper. A correlation between clinical severity and NfL levels in CSF could not be shown.

Körtvelyessy et al. investigated NfL levels in CSF and serum in 21 GBS patients using an ELISA. Levels were compared to 19 controls with non-neurological diseases [107]. In CSF, NfL levels (mean = 7623,149–50,000 pg/mL) were significantly higher than in controls (mean = 1114, 545–1957 pg/mL, *p* = 0.02). GBS disability scale score correlated with CSF NfL (*p* = 0.005). To differentiate between NfL of central or peripheral nerval origin, a ratio of CSF NfL and serum NfL was formed. A ratio of 12.8 was used as a cutoff. Patients with a lower ratio had axonal or mixed patterns in nerve conduction studies and a slightly higher clinical affection. Patients with a higher NfL-ratio showed a demyelinating pattern.

In conclusion, Nfs seem to be a promising marker not necessarily for diagnosis of GBS, but for differentiation between subtypes and even prediction of long-term outcome especially in axonal subtypes. Further investigations are, however, needed, especially studies with larger cohorts of patients, which may potentially answer further questions, e.g., which Nf would be the more promising biomarker. A more complicated question would be whether this biomarker could identify patients at risk of severe disease course, select them for a more aggressive treatment, or ameliorate the clinical outcome.

#### 4.1.2. Sphingomyelin and Lipidomics

Sphingomyelin is a sphingophospholipid typically found in the membrane of the Schwann cells that form the myelin sheath of peripheral nerves [108]. Its investigation therefore may be interesting in demyelinating neuropathies. Capodivento et al. found elevated sphingomyelin (SM) CSF levels in 14 patients with demyelinating neuropathies (*p* = 3.81 × 10^−8^) compared to 15 controls with ONDs [109]. To rule out a bias by BBB dysfunction, SM levels in demyelinating neuropathies were compared to 13 controls with obvious BBB dysfunction. Even in this comparison, a significant elevation could be shown (*p* = 1.34 × 10^−7^). The SM cut-off for optimum sensitivity and specificity was 0.00118 nmol/μL. Elevated SM correlated negatively with conduction velocity, which is a marker of demyelination, in 12 patients. A correlation with an axonal pattern in nerve conduction studies could not be shown.

To further investigate this novel biomarker, another study was conducted by Capodivento et al. in 2021. Twelve patients with the demyelinating subtype of GBS showed significantly elevated SM CSF levels (*p* < 0.0001) compared to patients with other neurological diseases [110]. Some overlap between the ranges of SM levels was found, but in the comparison of demyelinating and axonal GBS this was less of a problem. In axonal GBS, no significant difference from the OND group could be found. A cut-off value of 0.9819 pmol/μL was calculated as a marker of demyelination, which is similar to the aforementioned cut-off value. In demyelinating GBS, the SM level correlated with the GBS disability scale (r = 0.8877, *p* = 0.003), Overall Neuropathy Limitation Scale (r = 0.5997, *p* = 0.0426) and MRC sum score (R = −0.606, *p* = 0.0405).

Péter et al. investigated markers of demyelination through a shotgun lipidomic mass spectrometry-based approach. A total of 19 patients with GBS were compared to 34 controls with non-demyelinating disorders [111]. In total, 222 different lipid species could be identified. A four-fold elevated lipid content of CSF was found in GBS patients (*p* = 2 × 10^−9^). All lipid classes were elevated, but to a different extent. Cholesteryl esters, phosphatidylcholine, and SM showed the biggest differences. The most elevated species were plasma derived. The concentration of several plasma-derived lipids correlated with the F-score. A lower relative abundancy of these plasma-derived species compared to brain-derived species correlated with clinical recovery. Plasma infiltration into the CSF seemed to play the main role.

These studies show the possible value of SM as a biomarker in demyelinating GBS. Especially interesting is the correlation with clinical severity. Further investigations, especially with larger cohorts, are needed.

#### 4.1.3. Proteins

##### Cystatin C

Cystatin C is currently widely used in the clinic to assess the glomerular filtration rate [112] and was found to be influenced by several other factors besides renal function [113]. A shotgun proteomic approach by Yang et al. in 2008 and 2009 found cystatin C levels in the CSF of eight GBS patients significantly decreased compared to controls with headaches (*p* = 0.001). Cystatin C was significantly lower in GBS than in controls (*p* < 0.001) [114,115]. Another study by Li et al. showed a 1.05-fold decrease [116]. Those studies used a 2-dimensional gel electrophoresis followed by MALDI-TOF mass spectrometry. In the studies by Yang, an additional ELISA was performed to quantify cystatin C levels. A correlation with outcome, clinical severity, and pattern in nerve conduction studies, however, was not found to be present. Therefore, cystatin C as a marker for GBS seems to need further investigation due to the fact that only semiquantitative approaches have been performed.

##### Transthyretin

Transthyretin is a plasma protein predominantly synthesized in the liver. Until now, there are more than 60 mutations known to cause systemic amyloidosis [117]. In routine diagnostics in serum, transthyretin is considered a negative acute phase protein that is decreased during systemic inflammation. The CSF level of transthyretin, which is also called prealbumin, was investigated by several studies as a biomarker for GBS. Jin et al. investigated the proteomic profile of CSF in five GBS patients by 2-dimensional gel electrophoresis followed by MALDI-TOF mass spectrometry [118]. A significant reduction in transthyretin levels compared to controls with other neurological disorders was found. A study by Chiang et al. measured CSF transthyretin level in 20 GBS patients and in patients with other neurological diseases using an ELISA [119]. GBS patients had a significantly higher transthyretin level (5.57 ± 0.49 mg/dL) than the control group (2.76 ± 0.19 mg/dL, *p* = 0.05). A correlation to the F-score could not be shown. Zhang et al. tested transthyretin CSF levels in 19 GBS patients with an ELISA approach [120]. In comparison to levels in patients with other neurological diseases, levels in GBS were elevated (GBS: 2.14 ± 0.11 mg/dL, OND: 1.49 ± 0.17 mg/dL, *p* < 0.05). A correlation to functional scores was not found. In conclusion, transthyretin as a biomarker for GBS needs further investigation due to the contradictory findings with both increased and decreased values. Because of the missing correlation to clinical scores, its value seems to be subordinate compared to Nf and SM.

##### Haptoglobin

Chang et al. identified potential biomarkers through 2-dimensional gel electrophoresis and MALDI-TOF mass spectrometry in the CSF of 24 patients with GBS [121]. Haptoglobin, which is a protein that has the capacity to bind hemoglobin and is usually seen as a marker of inflammation and hemolysis, was found to be elevated. As a consequence, further quantification was performed by ELISA. CSF levels of haptoglobin in the GBS patients (12.44 ± 2.70 mg/mL) were significantly higher than in controls (1.44 ± 0.35 mg/mL, *p* = 0.05). Even a significant difference when compared to chronic inflammatory demyelinating polyneuropathy (CIDP) could be shown (2.82 ± 0.83 mg/mL, *p* = 0.05). A correlation with the F-score could not be shown.

The aforementioned paper by Jin et al. found a significant elevation in the CSF of five GBS patients by 2-dimensional gel electrophoresis followed by MALDI-TOF mass spectrometry. A further quantification or correlation with clinical data was not performed. A similar result with related methods was found by Lehmensiek et al. [122]. Through a 2-dimensional gel electrophoresis followed by MALDI-TOF mass spectrometry in six GBS patients, a significant upregulation of haptoglobin in comparison to 12 controls with tension-type headache could be found. In the above-mentioned study by Zhang et al., haptoglobin was found to be increased using an ELISA in 19 patients with GBS (GBS: 2.54 ± 0.46 mg/dL, OND: 0.48 ± 0.07 mg/dL, *p* < 0.001). A correlation with the F-score was not found. Li et al. found a 1.66-fold overrepresentation (*p* < 0.001) of haptoglobin in the above-mentioned study. No significant difference between AIDP and AMAN was found. In conclusion, haptoglobin seems to be a potential biomarker for GBS. A shortcoming, compared to other markers is the missing correlation to clinical severity. However, further investigations, especially due to the small cohort size, seem necessary.

##### Tau

Tau is a protein expressed in neurons of both the central and peripheral nervous system. Although the abnormal aggregation of tau into so-called neurofibrillar tangles is one of the major hallmarks in a variety of neurodegenerative diseases, its definite function remains elusive [123]. Due to its close relation to neurodegeneration, several studies have examined its CSF level in GBS, potentially revealing a disturbed tau-pathology.

Süssmuth et al. evaluated tau CSF levels using a sandwich ELISA in 61 patients with a multitude of neurological disorders, including five patients with GBS [124]. Four of those patients had tau levels below the detection limit of 59 ng/L, the fifth patient had a level of 106 ng/L. In comparison with the other groups of diseases, the GBS group had the lowest tau levels. Jin et al. correlated CSF tau levels in 26 GBS patients with clinical severity and outcome, measured by the F-score at nadir and after six months [125]. An F-score > 2 after six months was considered a poor outcome. A correlation between higher tau levels in GBS and a poor outcome was found (*p* < 0.01).

In the above-mentioned study by Petzold et al., CSF tau levels in 38 GBS patients were measured using ELISA. When compared to controls with ONDs, a significant difference in tau levels was not found. However, a correlation of tau levels with F-score as a measurement for the outcome was found (R = 0.47, *p* = 0.008). Wang et al. measured tau CSF levels in 43 patients with GBS using a sandwich ELISA and compared them to a group of patients with other neurological diseases [103]. A significant increase in CSF tau levels (*p* < 0.001) was found in both the AIDP and AMAN subgroups. A correlation of tau levels in CSF was found with the clinical severity in AMAN (*p* = 0.025, R = 0.698).

The investigations of tau levels in GBS seem contradictory. A possible explanation are the different study populations. Süssmuth and Petzold probably performed studies on a central European population, whereas the studies of Wang and Jin were performed on an Asian population, in which an axonal subtype of GBS is much more frequent. Therefore, further investigations that include differentiation between subtypes are needed.

##### Cytokines

As mentioned above, cytokines are essential mediators in inflammatory processes and are secreted by various immune cells like T and B lymphocytes, endothelial cells, mast cells, and macrophages [44,45,46] and are therefore of high interest when examining patients with inflammatory diseases. Thus, Breville et al. measured IL-8 CSF levels in four patients with GBS and compared them to levels in five patients with CIDP and four patients with non-inflammatory polyneuropathies [67]. A significant elevation of IL-8 was found in GBS (mean = 106 pg/mL) in comparison to CIDP (mean = 43 pg/mL, *p* = 0.003) and non-inflammatory polyneuropathies (mean = 28 pg/mL, *p* = 0.02). Sainaghi et al. tested the CSF of nine GBS patients, eight CIDP patients, and seven controls for concentrations of 50 different cytokines [68]. CXCL10 level was found to be higher in GBS than in CIDP and higher in CIDP than in controls. CCL7 level was higher in both neuropathies than in controls and higher in CIDP than in GBS. IL-8 and IL-1ra levels were higher in GBS than in CIDP and controls (*p* < 0.002).

The investigations of cytokines show promising results in the differentiation of GBS and CIDP, which is a highly relevant question due to its different therapeutic approaches. However, further investigations in greater cohorts seem necessary.

## 5. Multifocal Motor Neuropathy

MMN is an inflammatory disease caused by a focal destruction of the myelin sheath of the lower motor neurons. Clinically, it is manifested by weakness and atrophy of the innervated muscles. MMN is an important differential diagnosis compared to motor neuron diseases like lower motor neuron predominant ALS and PMA due to its promising treatment options compared to those degenerative diseases [26]. Typical findings are a conduction block in nerve conduction studies and the presence of anti-G_M1_ antibodies. However, the typical antibody is only present in about every second patient, so there is a need for more biomarkers. Furukawa et al. investigated the CSF levels of 28 different cytokines using a multiplex bead array assay and ELISA in 12 patients with MMN, eight with PMA, 26 with ALS, and 10 control patients with other neurological disorders [30]. In PMA, an elevation of IL-10, FGF-2, G-CSF, and VEGF could be shown in comparison to MMN. In the comparison of ALS with MMN, an elevation of IL-4, IL-17, FGF-2, and G-CSF levels was found (see also Table 1). In conclusion, the authors present a promising approach to solve this highly relevant question. However, further investigations in a larger cohort of patients seem necessary to assess their suitability as potential biomarkers.

## 6. Neuroborreliosis

Neuroborreliosis is caused by *Borrelia burgdorferi s. l.* The spirochetal infection causes a variety of symptoms including skin manifestations, lymphocytic meningitis, and peripheral neuropathies. A diagnosis of neuroborreliosis is usually supported by an elevated cell count in CSF and a ratio of IgG anti-Borrelia antibodies greater than two in comparison between CSF and serum. It was found that the criterion of elevated CSF antibodies only has a sensitivity of 75% and a specificity of 97% [126]. Therefore, there is a need for further biomarkers. Pietikäinen et al. investigated the levels of 49 different cytokines in 43 patients with neuroborreliosis [69]. A magnetic bead suspension array and ELISA was used to assess the levels of 49 cytokines. A multitude of cytokines was elevated in the CSF of neuroborreliosis patients. The best differentiation between groups was made by chemokine CXCL13 (see also Table 1). In a follow-up survey after antibiotic treatment, decreased levels of CXCL13 could indicate its value as a marker of treatment response. CXCL13 seems to be a potential candidate as a biomarker for diagnosis and control of treatment success in neuroborreliosis. Still, further investigations with larger cohorts are necessary. The presented study tested chemokine levels in patients that were diagnosed with the abovementioned criterion of an elevated antibody ratio with a sensitivity of only 75%. The investigation of CXCL13 in patients with clinically suspected neuroborreliosis but an antibody index below 2 would be of great interest.

## 7. Spinal Muscular Atrophy

Spinal muscular atrophy (SMA) is an autosomal-recessively inherited disease characterized by a severe loss of lower motor neurons due to low levels of the survival motor neuron (SMN) protein [127]. Two genes varying by only one nucleotide, *SMN1* and *SMN2*, encode SMN [128]. SMA patients carry mutations in both alleles of *SMN1*, leading to a low abundance of SMN protein. The severeness of the SMA phenotype correlates with the number of *SMN2* copies the patient has [129]. Five different types of SMA are clinically characterized based on the age of symptom onset and motor impairment, being ranked in severeness from type 0 (prenatal onset; respiratory failure at birth) to type 4 (onset in adulthood, ambulatory) [130]. Currently, there are three different drugs approved by the FDA for treatment of SMA [131]: risdiplam, which modulates the splicing of *SMN2*; onasemnogene abeparvovec, a non-replicating adeno-associated virus capsid delivering a *SMN1* copy; and nusinersen, which is an antisense oligonucleotide increasing the protein level of SMN by modulating the splicing of *SMN2*-mRNA [132]. Until now, treatment with all three drugs is very expensive and treatment efficiency cannot be monitored besides with clinical assessments, as suitable biomarkers are missing. Because nusinersen has to be administered by intrathecal injection, CSF samples are often collected before the administration and CSF biomarkers would thus be very valuable. Therefore, we focused on studies that evaluated CSF parameters and biomarkers in SMA patients following a nusinersen treatment.

### 7.1. Routine CSF Parameters in SMA

Besides protein biomarkers, there are different routine parameters often evaluated in CSF samples of SMA patients. Total protein concentration in CSF is regularly determined as well as the quotient of albumin levels in CSF and serum (Q_Alb_). Both values are measures of an intact blood–CSF barrier [133]. Both parameters were found to be increased in several studies in patients with SMA type 2, 3, or 4 at until up to 10 months of treatment compared to baseline values [134,135,136]. Total protein levels in CSF were not increased in SMA patients compared to healthy controls and changes in total protein levels during the time of observation did not correlate with changes in HFMSE score, which is a common criterion for motor function in SMA patients [134]. In one of the aforementioned studies, the researchers suggested that the slight changes in total protein and Q_Alb_ values were caused by the repeated intrathecal administration of nusinersen and the repeated lumbar punctures [135]. Furthermore, it is known that Q_Alb_ value and total protein level in CSF are age-related [133,137], stressing that both parameters are not suited as biomarkers for treatment monitoring in SMA type 2, 3, or 4 patients.

Another routine parameter, often investigated in CSF samples from SMA patients, is the number of cells per milliliter CSF. Depending on the study, different numbers of cells (four or five per μl) are concerned as “increased”. The researchers use the cell count as an indicator of possible inflammation caused by the repeated injection of nusinersen. The number of cells is commonly measured via manual counting after Pappenheim staining is performed. In the studies included in this review, only a few patients showed an increased number of white blood cells in the CSF and the few findings were not thought to be treatment-related [134,135]. Nevertheless, a very interesting finding concerned macrophages, which appeared in the CSF of nusinersen treated patients after the first treatment [138,139]. Two independent research groups reported about the characteristic inclusions inside the macrophages. Both groups could only hypothesize about the content of these inclusions and further investigations are required. Strikingly, the macrophages with these characteristic inclusions could only be found in SMA patients after nusinersen treatment and could not be observed in CSF samples of any healthy control patients. These findings stress that cells inside the CSF may be affected by the nusinersen treatment and proteomic investigation of these cells might provide further insights into disease progression and treatment efficiency.

### 7.2. Protein Biomarkers in SMA

In the past, several research groups have evaluated proteins as potential biomarkers for the progression of SMA over the course of nusinersen treatment. As reports about protein biomarker levels in response to nusinersen treatment are still quite rare, we also included proteins only investigated in a single study. We focused on research about aβ-peptides, tau protein, glial fibrillary acid protein (GFAP), and Nfs (detailed information on assessed studies can be found in Table 5). These proteins are all known to be related to neurodegenerative processes in other diseases like ALS [140], MS [141], Alzheimer’s disease (AD) [142], or Parkinson’s disease [143]. Furthermore, tau and Nfs like NfL and pNfH were already covered in this review, as their suitability as CSF biomarkers was also evaluated for ALS and GBS. This already suggests that they are not very specific to SMA progression.

Aβ-peptides 40 and 42 are often investigated as potential biomarkers in AD, as they derive from the amyloid precursor protein (APP) and are a major component of the AD-plaques. In a recently published study, the protein levels of aβ 40- and aβ 42 of eight patients affected by SMA type 2 or 3 were monitored over 14 months of nusinersen treatment [144]. While the level of Aβ 40 did not change, the level of Aβ 42 increased significantly in the CSF compared to baseline values. As a control group was missing, the suitability of aβ 42 as a biomarker for nusinersen treatment effectivity in SMA patients cannot be evaluated.

An additional protein known to be associated with AD progression and investigated as a potential biomarker for the monitoring of SMA treatment is the microtubule-associated protein tau [145]. For SMA 1 patients, a significant decrease in tau and phosphorylated tau (ptau) protein levels was observed in CSF samples after treatment with nusinersen, which did correlate with an improvement in CHOP-Intend score [146,147]. However, similar findings could not be verified in another study, which included 11 SMA type 3 patients [148]. Tau levels remained stable over the course of treatment, thus the researchers stated that tau levels in CSF might not be suitable for treatment monitoring in SMA type 3 patients. Due to the faster disease progression, tau protein levels in CSF may be suitable for treatment monitoring in SMA type 1 patients.

GFAP is a well-known marker for astrocytes in the CNS. Therefore, it is often used to investigate neuroinflammatory processes. GFAP level in CSF samples of 12 SMA type 1 patients were found to be significantly increased at the start of the investigation period compared to healthy controls [146]. Although a slight decrease in GFAP levels in CSF was observed, this change did not correlate with the improvement of the HFMSE score in the nusinersen treated SMA patients. A potential reason for this observation may be an age-related change in the GFAP level in CSF, as it was reported in a study including healthy volunteers [149]. Thus, GFAP can be stated as not suitable as a monitoring biomarker of nusinersen treatment.

As for GFAP, an age-related change in the protein levels in CSF was also reported for Nfs [149]. In general, Nfs are known to be essential for the cytoskeleton of neurons [150]. There are several studies on Nfs in SMA patients, most of them on SMA type 1 or SMA type 2/3 patients [134,136,147,148,149,151,152,153]. The overall trend is that Nf levels are better suited to monitor the treatment effect in SMA type 1 patients than in SMA type 2/3 patients, potentially due to the faster disease progression. In a study including 12 SMA type 1 patients, a decrease in the Nf levels in CSF correlated with an increase in CHOP-Intend score [146]. In all studies, Nf protein levels were quantified using ELISA kits, which are known to vary in sensitivity. Strikingly, in one study it was stated that out of 25 CSF samples, Nf protein levels were below the lower limit of quantification (LLOQ) of the used ELISA kits in 21 samples [153]. This finding stresses the need for a more accurate and sensitive method for quantification of Nf protein levels in CSF of SMA patients, potentially a targeted mass-spectrometry approach.

Surprisingly, there is currently only one study published in which the proteome in CSF of SMA patients was investigated using mass spectrometry [134]. Unfortunately, this study did not include proteomic analyses of cells in CSF samples of SMA patients. CSF samples from 10 SMA patients, one diagnosed with SMA type 2 and nine diagnosed with SMA type 3, were included in the study and CSF proteomes were compared to 10 age- and sex-matched controls. CSF samples were analyzed via LC-MS/MS at baseline and after 10 months of nusinersen-treatment and protein levels were compared. In SMA patients, not a single protein was of different abundance after 10 months of nusinersen treatment. The researchers hypothesized, that the observed effect may occur due to the limited number of patients, the restricted time frame, and the slow disease progression of SMA type 2 and 3. Thus, further mass spectrometric investigations on CSF samples of SMA type 1 patients or with a higher number of patients of different SMA types may provide promising insights and reveal candidates as biomarkers for nusinersen treatment response. As the results of cytological investigations showed that cells in CSF may be affected by nusinersen treatment, the proteome of these cells should also be investigated.

**Figure 1:**
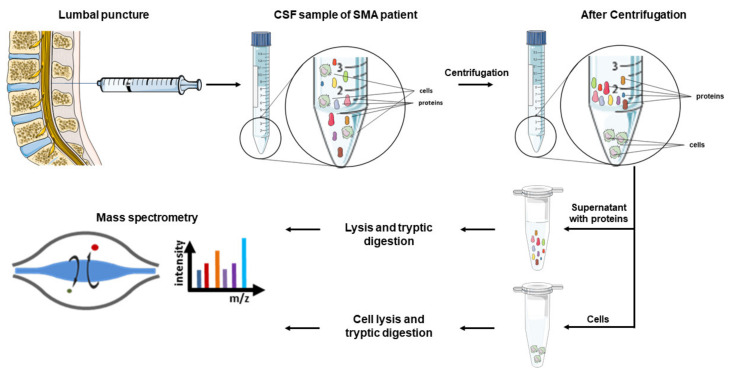
Potential workflow for a mass spectrometric analysis of CSF samples obtained from SMA patients. CSF samples of SMA patients are obtained in the context of intrathecal nusinersen treatment. After collection, proteins and cells contained in the CSF samples are separated via centrifugation and separately prepared for mass spectrometric analyses. The used schematics in this figure were partly provided by Servier Medical art (https://smart.servier.com/, accessed date: 17 November 2020). Servier Medical Art by Servier is licensed under a Creative Commons Attribution 3.0 Unported License.

How such a study could be set up is graphically summarized in Figure 1. Therefore, CSF samples of SMA patients, obtained via lumbar punctures in the context of nusinersen treatment, could be used. The proteins and cells present in the obtained CSF samples then get separated by centrifugation and split into two fractions, one being the pellet consisting of the cells, the other being the supernatant containing the proteins. The resulting samples should then be prepared for mass spectrometric measurements by lysis and a tryptic digestion.

## 8. Conclusions

In this review, a variety of neuromuscular diseases have been discussed, involving the degeneration of motor neurons. The review focused on the utilization of CSF as a suitable bodyfluid for the detection of biomarkers, not only to identify the disease, but also to determine disease progression. The most promising biomarkers so far are Nfs, specifically the combination of NfL and pNfH for the identification and determination of disease progression in ALS, as levels measured by ELISA are significantly higher than in other neurological diseases and ALS mimics. Furthermore, this method is already routinely used in the clinic. However, it must be mentioned that all measurements are subject to strong fluctuations and that defined criteria for CSF withdrawal and ELISA assays must be formulated to ensure a reproducible detection. An interesting alternative for ALS diagnostics could be the quantification of TDP43, especially if the hyperphosphorylated form is included. To ensure robust quantification of these biomarkers, alternative methods besides the classical ELISA should be considered, especially the enrichment of pathological hyperphosphorylated forms and their absolute quantification using targeted mass spectrometry. In the group of peripheral neuropathies, a multitude of biomarkers has been investigated. In our opinion, for GBS the most promising candidates are SM for AIDP and Nf for AMAN. In Lyme neuroborreliosis and MMN, the examination of cytokines seems promising not only to ease the diagnosis of neuroborreliosis, but also to identify MMN as an important, treatable differential diagnosis to ALS. For SMA, no convenient biomarker in CSF can be reported that can be used for treatment monitoring different SMA types. Especially for SMA types 2 and 3, biomarkers for treatment monitoring are needed, as the benefit of the treatment needs to be ensured due to the immense costs of all FDA-approved drugs.

## Figures and Tables

**Table 1:** Cytokines evaluated in the four different studies. For a better comparison between diseases, the table further displays the results of the later mentioned studies investigating cytokines in GBS and neuroborreliosis. Significance between the groups is indicated with a √ for significant, x for not significant, - for not evaluated, and n. e. for not expressed (lower than the assay cut off). Abbreviations: ALS = amyotrophic lateral sclerosis, MMN= multifocal motor neuropathy OND = other neurological diseases, PMA = progressive muscular atrophy, GBS = Guillain-Barré syndrome, CIDP = chronic inflammatory demyelinating polyneuropathy, NIP = noninflammatory polyneuropathy, LNB = Lyme neuroborreliosis, MS = multiple sclerosis, TBE = tick-borne encephalitis.

Cytokines	Tateishi et al.	Mitchell et al.	Furukawa et al.					Italiani et al.	Breville et al.		Sainaghi et al.			Pietikäinen et al.		
	ALS/ONDs	ALS/ONDs	ALS/ONDs	ALS/MMN	PMA/ONDs	PMA/MMN	MMN/ONDs	ALS/ ONDs	GBS/CIDP	GBS/NIP	GBS/CIDP	GBS/Control	CIDP/Control	LNB/Control	LNB/MS	LNB/TBE
IL-1α	-	-	-	-	-	-	-	-	-	-	-	-	-	x	√	x
IL-1β	√	x	x	x	x	x	x	x	-	-	n. e.	n. e.	n. e.	√	√	x
IL-1ra	x	x	x	x	√	x	√	x	-	-	√	√	x	√	√	x
IL-2	x	√	x	x	x	x	x	-	-	-	n. e.	n. e.	n. e.	n. e.	n. e.	n. e.
IL-2ra	-	-	-	-	-	-	-	-	-	-	x	x	x	x	x	x
IL-3	-	-	-	-	-	-	-	-	-	-	-	-	-	√	√	x
IL-4	x	x	√	√	x	x	x	-	-	-	n. e.	n. e.	n. e.	√	x	√
IL-5	x	x	x	x	x	x	x	-	-	-	n. e.	n. e.	n. e.	n. e.	n. e.	n. e.
IL-6	x	√	x	x	x	x	x	-	x	x	x	x	x	√	√	√
IL-7	√	x	√	x	√	x	x	-	-	-	n. e.	n. e.	n. e.	√	√	√
IL-9	√	x	x	x	x	x	x	-	-	-	x	x	x	√	√	x
IL-10	x	x	x	x	√	√	x	-	-	-	n. e.	n. e.	n. e.	√	√	√
IL-12	√	x	x	x	x	x	x	-	-	-	x	x	x	√	√	√
IL-13	x	x	x	x	x	x	x	-	-	-	n. e.	n. e.	n. e.	√	√	x
IL-15	x	√	x	x	x	x	x	-	-	-	x	x	x	x	x	√
IL-16	-	-	-	-	-	-	-	-	-	-	x	x	x	√	√	x
IL-17	√	√	√	√	√	x	x	-	-	-	n. e.	n. e.	n. e.	√	x	√
TNF-α	√	x	x	x	x	x	x	-	x	x	n. e.	n. e.	n. e.	√	√	√
TNF-β	-	-	-	-	-	-	-	-	-	-	-	-	-	x	x	x
IFNα-2	-	-	-	-	-	-	-	-	-	-	x	x	x	√	x	x
IFN-y	√	x	x	x	x	x	x	-	-	-	n. e.	n. e.	n. e.	√	√	√
CCL2	√	√	x	x	x	x	x	-	-	-	x	√	√	x	x	x
CCL3	x	√	x	x	x	x	x	-	-	-	n. e.	n. e.	n. e.	√	√	x
CCL4	√	√	x	x	x	x	x	-	-	-	x	x	x	-	-	-
CCL5	√	x	x	x	x	x	x	-	-	-	n. e.	n. e.	n. e.	√	√	x
CCL7	-	-	-	-	-	-	-	-	-	-	√	√	√	n. e.	n. e.	n. e.
CCL11	√	x	√	x	√	x	x	-	-	-	n. e.	n. e.	n. e.	√	√	√
CCL27	-	-	-	-	-	-	-	-	-	-	x	√	√	√	√	x
CXCL1	-	-	-	-	-	-	-	-	-	-	x	x	x	√	√	x
CXCL8	√	x	x	x	x	x	x	-	√	√	√	√	x	√	√	x
CXCL9	-	-	-	-	-	-	-	-	-	-	x	√	√	√	√	x
CXCL10	√	x	x	x	x	x	x	-	-	-	√	√	√	√	√	x
CXCL 12	-	-	-	-	-	-	-	-	-	-	x	√	√	-	-	-
CXCL 12α	-	-	-	-	-	-	-	-	-	-	-	-	-	√	√	x
CXCL13	-	-	-	-	-	-	-	-	-	-	-	-	-	√	√	√
G-CSF	√	√	√	√	√	√	x	-	-	-	x	x	x	√	√	√
GM-CSF	x	√	x	x	x	x	x	-	-	-	x	x	x	√	√	x
M-CSF	-	-	-	-	-	-	-	-	-	-	x	x	x	x	x	x
FGF-2	x	√	√	√	√	√	x	-	-	-	x	x	x	√	√	√
PDGFbb	x	x	√	x	√	x	x	-	-	-	x	x	x	√	√	x
VEGF	√	√	x	x	√	√	x	-	-	-	x	√	√	√	√	x
β2M	-	x	-	-	-	-	-	-	-	-	-	-	-	-	-	-
TNFR1	-	-	x	x	√	x	x	-	-	-	-	-	-	-	-	-
sIL-1R2	-	-	-	-	-	-	-	x	-	-	-	-	-	-	-	-
IL-33	-	-	-	-	-	-	-	x	-	-	-	-	-	-	-	-
sIL-1R4	-	-	-	-	-	-	-	x	-	-	-	-	-	-	-	-
IL-37	-	-	-	-	-	-	-	x	-	-	-	-	-	-	-	-
IL-18	-	-	-	-	-	-	-	√	-	-	x	x	x	√	√	x
IL-18BP	-	-	-	-	-	-	-	√	-	-	-	-	-	-	-	-
ICAM-1	-	-	-	-	-	-	-	-	-	-	x	√	√	-	-	-
VCAM1	-	-	-	-	-	-	-	-	-	-	x	√	√	-	-	-
SCF	-	-	-	-	-	-	-	-	-	-	√	x	√	√	√	x
LIF	-	-	-	-	-	-	-	-	-	-	x	x	x	x	x	x
MIF	-	-	-	-	-	-	-	-	-	-	x	x	x	x	√	x
SCGF-b	-	-	-	-	-	-	-	-	-	-	x	x	x	x	√	x
HGF	-	-	-	-	-	-	-	-	-	-	√	x	√	x	x	x
TRAIL	-	-	-	-	-	-	-	-	-	-	x	x	x	√	√	x
β-NGF	-	-	-	-	-	-	-	-	-	-	-	-	-	√	√	x

**Table 2:** Information on cohort selection size and composition, body fluid, as well as used technique/antibody and detected TDP-43 levels among the assessed studies. Abbreviations: ALS = amyotrophic lateral sclerosis, FTLD = frontotemporal lobar degeneration, GBS = Guillain Barré syndrome, MS= multiple sclerosis, PD = Parkinson’s disease.

Paper	Cohort Size and Composition	Body Fluid	Marker	Method for TDP-43	Antibody	Results TDP-43 ng/mL
Kasai et al., 2009	30 ALS, 29 controls (13 controls, 16 disease controls)	CSF	TDP-43	Sandwich ELISA (Nunc MaxiSorp, Xat-bottom 96-well Black MicroWell plate, Roskilde, Denmark)	anti-TDP-43 monoclonal antibody, detection antibody, anti-TDP-43 rabbit polyclonal antibody (10782-2-AP, ProteinTech Group, Chicago, IL, USA), raised against a recombinant protein corresponding to residues 1–261 of human TDP-43 (H00023435-M01, clone 2E2-D3, Abnova Corporation, Walnut, CA, USA), detection antibody, anti-TDP-43 rabbit polyclonal antibody (10782-2-AP, ProteinTech Group, Chicago, IL, USA)	ALS: 6.92 +/− 3.71; Control: 5.31 +/− 0.94
Noto et al., 2010	27 ALS, 50 disease controls (15 PD, 15 MS, 20 GBS)	CSF	TDP-43	Sandwich ELISA (Nunc MaxiSorp, Xat-bottom 96-well Black MicroWell plate, Roskilde, Denmark)	anti-TDP-43 monoclonal antibody (H00023435-M01, clone 2E2-D3, Abnova Corporation, Walnut, CA, USA), detection antibody, anti-TDP-43 rabbit polyclonal antibody (10782-2-AP, ProteinTech Group, Chicago, IL, USA)	ALS: 29.5 +/− 15.5; Control PD: 19.7 +/− 6.6, Control MS: 13.7 +/− 9.0, Control GBS: 16.7 +/− 7.5
Hosogawa et al., 2014	13 ALS, 7 GBS	CSF	TDP-43	Sandwich ELISA (Nunc MaxiSorp, Xat-bottom 96-well Black MicroWell plate, Roskilde, Denmark)	monoclonal antibody, clone 2E2-D3 (Abnova Corp., Taipei, Taiwan), for capture and a rabbit polyclonal antibody (catalog code 10782-2-AP, ProteinTech Group Inc., Chicago, IL, USA) for detection.	ALS: 1.68 +/− 0.15; GBS: 1.05 +/− 0.13
Steinacker et al., 2008	15 ALS, 12 FTLD, 9 ALS + FTLD, 3 ALS + frontal disinhibition, 13 controls	CSF	TDP-43	Immunoblot	Affinity purified polyclonal rabbit antibody raised against amino acids 1 through 260 of recombinant TDP-43 (1:2000 and 1:10,000 to 1:1000; Proteintech Group Inc, Chicago, IL, USA), Monoclonal TDP-43 antibody clone 2E2-D3 specific for amino acids 205 through 25517 (1:1000; Abnova, Taipei City, Taiwan). Polyclonal rabbit antisera raised against N-terminus amino acids 6 through 24 or against C-terminus amino acids 396 through 414 of TDP-43 (1:5000 for both)	not applicable
Feneberg et al., 2014	9 ALS, 4 FTLD, 8 controls	CSF, Plasma and Brain	TDP-43	Immunoblot	Antibodies against different TDP-43 epitopes (N-terminus, C-terminus, and aa 205–222), against calnexin, GP Ib-V-IX and flotillin-1. Standard (human Jurkat cells and murine neuroblastoma (N2A) cells were used)	not applicable

**Table 3:** Information on cohort selection size and composition, body fluid, as well as used technique/antibody and detected neurofilament light chain (NfL) and phosphorylated neurofilament heavy chain (pNfH) levels among the assessed studies. Abbreviations: AD = Alzheimers disease, ALS = amyotrophic lateral sclerosis, CS = corticobasal syndrome, DC = disease controls, DLB = dementia with Lewy bodies, DM = disease mimics, DS = down syndrome, MCI = mild cognitive impairment, PD = Parkinson’s disease, PSP = progressive supranuclear palsy, PDLB = prodromal dementia with Lewy bodies.

Paper	Cohort Size and Composition	Body Fluid	Marker	Method for NfL, pNfH	Results pNfH pg/mL	Results NfL [pg/mL]
Poesen et al., 2017	220 ALS, 316 DC, 50 DM	CSF	NfL, pNfH	ELISA kits for pNfH (Biovendor, Brno, Czech Republic; RD191138300R, average test-retest variance of 6.8%) and NfL (UmanDiagnostics AB, Umea, Sweden; UD51001, average test-retest variance of 4.9%).	ALS: 2366 (114–18,089), DC: 289 (24–18,740) DM: 296 (24–7049)	ALS: 9427 (370–108,909). DC: 1790 (262–53,677), DM: 1407 (613–36,597)
Rossi et al., 2018	190 ALS, 130 mixed neurological diseases CTRL-1 (non-inflammatory neurological diseases), CTRL-2 (patients with acute/subacute inflammatory diseases and tumors)	CSF	NfL, pNfH	Single-batch ELISA kits for NF-L assays (iMyBioSource San Diego, USA and UmanDiagnostics AB, Umeå, Sweden). For pNF-ELISA kit from BioVendor Research and Diagnostic Product, Czech Republic)	ALS:1700 (760–3170), CTRL 1: 30 (0.00–320) CTRL 2: 820 (0.00–3470)	MyoBioSource kit: ALS: 2140 (1350–3300), CTRL 1: 2040 (1250–3390) CTRL 2: 3090 (1120–4590); UmanDiagnostics kit: ALS: 4700 (760–3170) CTRL 1: 300 (0.00–320) CTRL 2: 820 (0.00–3470)
Olsson et al., 2018	68 ALS, 75 controls, 114 patients with MCI, 397 AD, 96 FTLD, 41 PD, 19 PD with MCI, 29 with PD dementia, 33 LBD, 21 with CS, 20 with progressive supranuclear palsy (PSP)	CSF	NfL	In-house ELISA (2 NFL mouse monoclonal antibodies (NFL21 and NFL23))	not tested	ALS: 4185 (2207–7453), CTRL: 536 (398–777), MCI: 831 (526–1075), AD: 951 (758–1261), FTD: 1873 (830–2588), PD: 619 (526–840), PD MCI: 779 (464–1021), PD dementia: 981 (679–1722), DLB: 991 (695–2139), CBS: 1281 (828–2713), PSP: 1578 (1287–3104)
Delaby et al., 2020	46, ALS, 118 Controls, 116 AD, 47 DS, 56, FTD, 37 DLB, 26 PDLB, 26 CS, 12 PSP	CSF	NfL	ELISA kit (NF-light, UMAN DIAGNOSTICS, Umea, Sweden)	not tested	ALS: 2953 (1664–4250), CTRL: 411 (343–567), AD: 940 (765–1229), DS: 349 (196–464), DS + AD: 955 (664–1497), FTLD: 1240 (859–2378), DLB: 1135 (803–1321), PDLB: 934 (643–1094), CBS: 1637 (923–2797) PSP: 1422 (1034–1727)
Oeckl et al., 2016	Multicenter study (15 centers, 5 ALS patients each (150 ALS patients in total), DC (details on composition in each center can be found in the respective paper)	CSF	NfL, pNfH	ELISA (pNfH: BioVendor GmbH; NfL: IBL International), Two aliqouts per sample One analysed at Neurochemical Laboratory at the Department of Neurology in Ulm and the other at the department of metabolic biochemistry, Hôpitaux Universitaires Pitié Salpêtrière-Charles Foix, Paris.	ALS: 1773.2 (average centers, Ulm), CTRL 476.3 (average centers, Ulm), ALS: 1755.1 (average all centers, Paris), CTRL 288.8 (average centers, Paris)	ALS: 4148.6 (average all centers, Ulm), CTRL 284.2 (average from all centers, Ulm) ALS: 11,577.2 (average all centers, Paris), CTRL 1242.4 (average from all centers, Paris)
De Schaepdryver et al., 2017	85 ALS, 215 DC, 31 DM	CSF and Plasma	pNfH	ELISA for pNfH concentrations (Euroimmun AG, Lübeck, Germany). ELISA from Biovendor (RD191138300R, Brno, Czech Republic), from 83 patients with ALS and 213 controls were used from the previous publication, 9 to perform a paired method comparison with the IVD ELISA from Euroimmun.	ALS: 2451 (314–17,247); DC: 281 (20–13,669)	not measured

**Table 4:** Information on cohort selection size and composition, body fluid, as well as used technique/antibody and detected TDP-43 levels among the assessed studies. Abbreviations: AIDP = acute inflammatory demyelinating polyneuropathy, AMAN = acute motor axonal neuropathy, CIDP = chronic inflammatory demyelinating polyneuropathy, GBS = Guillain Barré syndrome, NfH = neurofilament heavy chain, NfL = neurofilament light chain, pNfH = phosphorylated neurofilament heavy chain, OND = other neurological disorders.

Paper	Cohort Size and Composition	Other Criteria	Marker	Method	Results
Petzold et al., 2006	23 GBS patients	Pattern, Outcome	NfH	ELISA	NfH level correlates with axonal degeneration and worse outcome
Petzold et al., 2009	38 GBS patients, 38 controls with other neurological conditions	Outcome	NfH	in-house developed ELISA.	Higher levels in group with poor outcome. NfH > 0.73 ng/mL predicts poor outcome
Wang et al., 2012	11 AIDP, 11 AMAN, 10 OND	Pattern, Outcome	pNfH	pNFH ELISA kits from Biovendor, Ostrava, Czech Republic;	pNfH higher in AMAN than in AIDP, Correlation between pNFH and outcome in AMAN
Dujmovic et al., 2016	3 GBS patients		NfH	ELISA	Higher levels of NfH seem to be associated with a poor outcome
Axelson et al., 2018	18 GBS patients	Outcome	NfL	ELISA	Poor outcome in patients with NfL levels over 10,000 ng/L
Mariotto et al., 2018	GBS (N = 5), MMN (N = 3), CIDP and variants (N = 12), anti-MAGneuropathy (N = 3), and non-systemic vasculitic neuropathy (N = 1)	Outcome	NfL	HD-1 immunoassay analyzer, Quanterix SimoaTM	No corellation between CSF NfL and outcome
Körtvelyessy et al., 2020	21 GBS patients, 19 controls	Pattern, Outcome, CSF/serum-ratio	NfL	ELISA	NfL significantly higher than in controls, GBSDS correlated with CSF-NfL, Ratio distinguishes between patterns

**Table 5:** Information on cohort selection size and composition, protein marker, method, as well as results of the assessed studies investigating protein biomarkers. Abbreviations: CSF = cerebrospinal fluid, GFAP = glial fibrillary acidic protein, NfL = neurofilament light chain, pNfH = phosphorylated neurofilament heavy chain, SMA = spinal muscular atrophy.

Paper	Cohort Size and Composition	Protein Marker	Methods	Results
Vagberg et al., 2015	53 healthy volunteers	NfL, GFAP	ELISA	NfL and GFAP levels in CSF correlated with age (NfL: rho = 0.870; GFAP: rho = 0.595; *p* < 0.001)
Olsson et al., 2019	12 patients with SMA type 1, 11 patients sampled for facial nerve palsy or to exclude meningitis or cerebellitis used as control	NfL, tau, GFAP	ELISA	mean baseline levels for all three markers were significantly increased in SMA 1 patients (NfL: 4598 ± 981 pg/mL vs. 148 ± 39 pg/mL, *p* = 0.001; tau: 939 ± 159 pg/mL vs. 404 ± 86 pg/mL, *p* = 0.02; GFAP: 236 ± 44 pg/mL vs. 108 ± 26 pg/mL, *p* = 0.02), NfL levels normalized <380 pg/mL at T4 or T5, tau and GFAP levels decreased over time, correlation with improvement in CHOP-INTEND score was observed for tau (rho = −0.85, *p* = 0.0008) and NfL (rho = −0.64, *p* = 0.03)
Totzeck et al., 2019	11 patients with SMA type 3	NfH, tau	ELISA	mean levels for tau and NfH remained stable within the reference range (tau: <290 pg/mL; NfH: <0.69 ng/mL)
Winter et al., 2019	1 patient with SMA type 1	NfL, pNfH, total tau, phosphorylated tau	not mentioned	NfL and pNfH level decreased under limit of detection at T4 resepctively T6, total and phosphorylated tau level decreased slightly
Wurster et al., 2019	9 patients with SMA type 2, 16 with SMA type 3, 25 control patients	NfL, pNfH	ELISA	Median NfL and pNfH levels did not differ significantly from controls at baseline or at T4
Faravelli et al., 2020	12 patients with SMA type 3, 9 control patients	NfL, pNfH	ELISA	NfL and pNfH levels were comparable between SMA patients and controls at baseline, NfL and pNfH levels decreased significantly after 6 months of treatment (*p* = 0.031 respectively *p* = 0.016)
Introna & Milella et al., 2021	8 patients with SMA type 2/3	aβ 40, aβ 42	solid-phase enzyme immunoassay	mean aβ 40 level remained stable during 420 days of Nusinersen-treatment (T0: 6437.5 ± 3201 pg/mL; T4: 6842.9 ± 1391.5 pg/mL, *p* = 0.498); mean aβ 42 level increased during treatment, significant at T2 and T4 (T0: 577.3 ± 227 pg/mL; T2: 634.6 ± 266 pg/mL, *p* = 0.012; T4: 891 ± 462.2 pg/mL, *p* = 0.018)

## Supplementary Materials

The following are available online at https://www.mdpi.com/article/10.3390/diagnostics11091522/s1, Supplementary Table S1, Supplementary Table S2, Supplementary Table S3.
