# Supplementary material for: CSF Diagnostics: A Potentially Valuable Tool in Neurodegenerative and Inflammatory Disorders Involving Motor Neurons: A Review
Source: Diagnostics (Basel). 2021 Aug 24;11(9):1522. doi: 10.3390/diagnostics11091522 (PMC8470638; doi:10.3390/diagnostics11091522)
Supplement: Supplementary file 1 [file diagnostics-11-01522-s001.zip › Supplementary Figure s1.pdf]

Supplementary Figure S1: Flow Diagram displaying the search strategy, keywords, as well as inclusion and exclusion criteria for A) Amyoptrophic Lateral Sclerosis (ALS), B) Peripheral neuropathies, C) Spinal muscular atrophy (SMA).

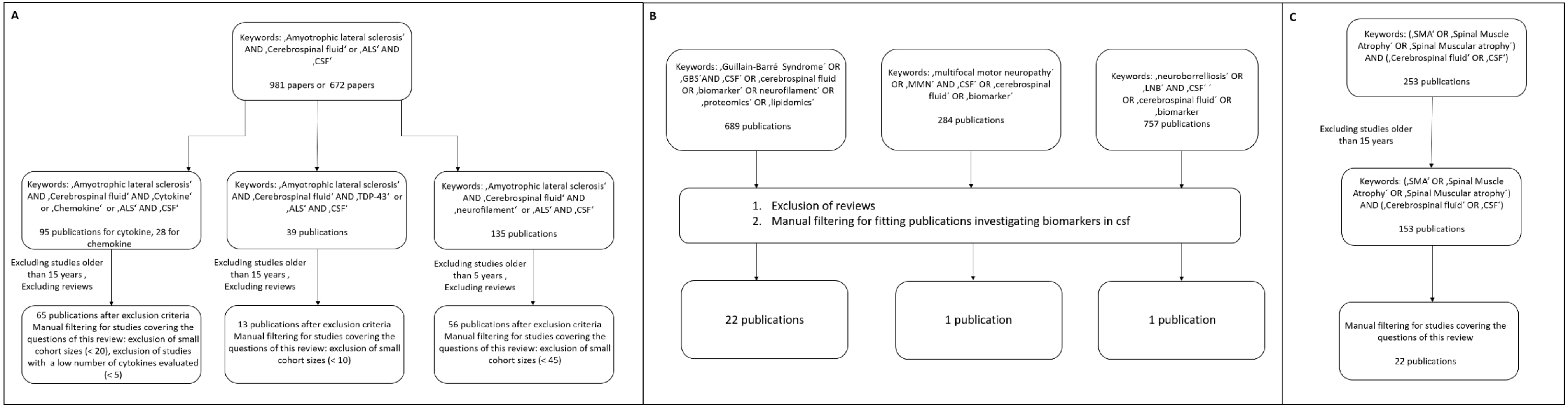
A
Keywords: ‚Amyotrophic lateral sclerosis' AND ‚Cerebrospinal fluid' or ‚ALS' AND ‚CSF'
981 papers or 672 papers
Keywords: ‚Amyotrophic lateral sclerosis' AND ‚Cerebrospinal fluid' AND ‚Cytokine' or ‚Chemokine' or ‚ALS' AND ‚CSF'
95 publications for cytokine, 28 for chemokine
Keywords: ‚Amyotrophic lateral sclerosis' AND ‚Cerebrospinal fluid' AND ‚TDP-43' or ‚ALS' AND ‚CSF'
39 publications
Keywords: ‚Amyotrophic lateral sclerosis' AND ‚Cerebrospinal fluid' AND ‚neurofilament' or ‚ALS' AND ‚CSF'
135 publications
Excluding studies older than 15 years , Excluding reviews
Excluding studies older than 15 years , Excluding reviews
Excluding studies older than 5 years , Excluding reviews
65 publications after exclusion criteria Manual filtering for studies covering the questions of this review: exclusion of small cohort sizes (< 20), exclusion of studies with a low number of cytokines evaluated (< 5)
13 publications after exclusion criteria Manual filtering for studies covering the questions of this review: exclusion of small cohort sizes (< 10)
56 publications after exclusion criteria Manual filtering for studies covering the questions of this review: exclusion of small cohort sizes (< 45)
B
Keywords: ‚Guillain-Barré Syndrome´ OR ‚GBS´AND ‚CSF´ OR ‚cerebrospinal fluid OR ‚biomarker´ OR neurofilament´ OR ‚proteomics´ OR ‚lipidomics´
689 publications
Keywords: ‚multifocal motor neuropathy´ OR ‚MMN´ AND ‚CSF´ OR ‚cerebrospinal fluid´ OR ‚biomarker´
284 publications
Keywords: ‚neuroborrelliosis´ OR ‚LNB´ AND ‚CSF´ ´ OR ‚cerebrospinal fluid´ OR ‚biomarker
757 publications
1. Exclusion of reviews
2. Manual filtering for fitting publications investigating biomarkers in csf
22 publications
1 publication
1 publication
C
Keywords: (‚SMA' OR ‚Spinal Muscle Atrophy´ OR ‚Spinal Muscular atrophy´) AND (‚Cerebrospinal fluid' OR ‚CSF')
253 publications
Excluding studies older than 15 years
Keywords: (‚SMA' OR ‚Spinal Muscle Atrophy´ OR ‚Spinal Muscular atrophy´) AND (‚Cerebrospinal fluid' OR ‚CSF')
153 publications
Manual filtering for studies covering the questions of this review
22 publications
